# A Multi-Ingredient Supplement Protects against Obesity and Infertility in Western Diet-Fed Mice

**DOI:** 10.3390/nu15030611

**Published:** 2023-01-25

**Authors:** Mats I. Nilsson, Linda May, Liza J. Roik, Matthew R. Fuda, Ashely Luo, Bart P. Hettinga, Adam L. Bujak, Mark A. Tarnopolsky

**Affiliations:** 1 Department of Pediatrics, McMaster University Medical Center (MUMC), Hamilton, ON L8N 3Z5, Canada; 2 Exerkine Corporation, McMaster University Medical Center (MUMC), Hamilton, ON L8N 3Z5, Canada

**Keywords:** obesity, infertility, subfertility, fat, liver, NAFLD, white adipose tissue, WAT, browning, mitochondria, PGC-1α, oxidative damage, inflammation, IL-1, TNF, CASP1

## Abstract

The Western diet (WD) predisposes to bodyweight gain and obesity and is linked to mitochondrial dysfunction, oxidative damage, inflammation, and multisystem disease, even affecting the reproductive organs, fertility, and pregnancy outcomes. In this study, we investigated the effects of multi-ingredient supplementation (MIS) with antioxidants, phytonutrients, and vitamins (‘Fertility Enhancer’; FE) on white adipose tissue (WAT) expansion, nonalcoholic fatty liver disease (NAFLD), and infertility in WD-fed C57BL/6J mice. Five-month-old male (M) and female (F) mice were fed a low-fat diet (LF) or a high fat/sucrose WD (HF) for six weeks, followed by six weeks of LF (3.64 kcal/g), HF (4.56 kcal/g), or HF combined with FE (4.50 kcal/g). A sub-set of animals were sacrificed at 12 weeks, while the remainder were harem-mated in a 1:2 male-to-female ratio, and singly housed during the gestational period. Two-way, factorial ANOVA analysis revealed a main effect of diet on bodyweight (BW), total body fat, % body fat, white adipose tissue mass, and liver lipid content (all *p* < 0.001), driven by the anti-obesogenic effects of the ‘Fertility Enhancer’. Similarly, a main effect of diet was found on PGC1-α mRNA levels (*p* < 0.05) and mitochondrial protein content (*p* < 0.001) in perigonadal WAT, with PGC1-α induction and higher complex II and complex III expression in FE vs. HF animals. Copulatory plug counts were higher in FE vs. HE couples (30% vs. 6%), resulting in more litters (4 vs. 0) and higher copulatory success (67% vs. 0%). Although the trends of all histology outcomes were suggestive of a benefit from the FE diet, only the number of atretic follicles and testicular mass were significant. Ovarian IL-1β mRNA induction was significantly attenuated in the FE group (*p* < 0.05 vs. HF) with CASP1 attenuation trending lower (*p* = 0.09 vs. HF), which is indicative of anti-inflammatory benefits of the ‘Fertility Enhancer.’ We conclude that supplementation with specific phytonutrients, antioxidants, and vitamins may have utility as an adjunctive therapy for weight management, fatty liver disease, and infertility in overweight and obese couples.

## 1. Introduction

Obesity is a multisystem disease linked to mitochondrial dysfunction, oxidative damage, and inflammation, primarily affecting critical organs or tissues involved in metabolism (e.g., fat, liver, pancreas, heart, and skeletal muscles) [1,2,3,4], thus significantly increasing comorbidity and mortality risks (e.g., cardiovascular disease (CVD), type 2 diabetes (T2DM), non-alcoholic fatty liver disease (NAFLD), and cancer) [5,6,7,8,9,10]. Excess food intake and weight gain also affect reproductive health by impairing gonadal function and fertility, thereby predisposing overweight and obese couples to reproductive system disorders and poor pregnancy outcomes couples [11].

Infertility is defined as the failure to achieve clinical pregnancy after one year of unprotected sex and affects an estimated 72.4 million couples worldwide (e.g., 10–15% of couples in industrialized nations) [12,13]. Reproductive health is considered a ‘couple concept’ because the fertility of both partners is important for a successful pregnancy [14]. It is estimated that about one third of all infertility cases are attributed to male factors, one third to female factors, and the remainder to a combination of both partners and unknown causes [15].

Epidemiological evidence suggests that obesity impairs both male and female fertility, reduces the efficacy of fertility treatments, and is linked to reproductive failure in a dose-dependent manner [11,16,17,18,19,20,21,22,23]. Specifically, obesity is linked to erectile dysfunction, hypoandrogenism, and altered spermatogenesis in men, and irregular menstruation, ovulatory dysfunction, hyperandrogenism, and increased time to pregnancy (TTP) in women, with a higher risk of birth defects, stillbirths, and infant obesity in neonates [17,18,24]. In a study of 3029 Dutch couples, van der Steeg and colleagues found that each unit increase in body mass index (BMI; bodyweight/height^2^) over 29 kg/m^2^ was associated with a 4% linear decrease in spontaneous pregnancy in females [25]. Another study involving 47,835 Danish couples demonstrated a dose-dependent relationship between increasing BMI and infertility in both men (odds ratio = 1.19; 95% CI 1.14–1.24) and women (odds ratio = 1.32; 95% CI 1.26–1.37) [14]. The authors concluded that there is a higher risk of infertility if both partners are overweight or obese, and emphasized that fecundity is a ‘couple concept’ with particular relevance to obesity [14].

Although the contribution of obesity to total infertility cases is largely unknown and the resultant economic consequences have not been conclusively demonstrated, Koning and colleagues developed a framework for estimating total cost per pregnancy in normal weight, overweight, and obese women [26]. Based on available systematic reviews and large-scale studies, the authors concluded that the total pregnancy cost was 50% to 85% higher in overweight and obese women, respectively, and exceeded €10,000 in some cases. Although the framework did not specifically account for the cost of ovulatory stimulating drugs (e.g., gonadotrophins), it is well known that higher dosages are required for overweight individuals, thus strengthening the argument for an increased cost per pregnancy in obese women [27]. Collectively, these data suggest that obesity may be associated with impaired fertility, poor reproductive outcomes, and increased cost per clinical pregnancy; likely contributing significantly to the global burden of infertility.

The multi-factorial origins of obesity are complex; however, an imbalance between energy intake and energy expenditure leading to energy surplus is considered the main reason for weight gain. Excess food intake and high fat/sucrose diets, such as Western diets (WD), predispose one to obesity, characterized by an expansion of white adipose tissue (WAT), elevated free fatty acids (FFA) levels, systemic inflammation (pro-inflammatory cytokines; IL-6, TNFα, IL-1, and MCP-1), mitochondrial dysfunction, oxidative stress, insulin resistance (IR), and ectopic lipid deposition in insulin-sensitive tissues such as liver [1,2,3,4]. Secondarily, other systems may be affected, including the neuroendocrine and reproductive systems, causing hypothalamic-pituitary-adrenal axis (HPA) dysregulation, oxidative damage, and inflammation, thereby impairing gonadal function and fertility.

Considering the complexity of obesity and associated conditions, a multi-treatment approach involving behavior and lifestyle modifications (caloric restriction, dietary changes, and physical activity (PA)), pharmacotherapy (regulation of appetite and metabolism), and/or surgical intervention (bariatric surgery) may be necessary [28]. In general, when the behavioral approach is insufficient, anti-obesity drugs and bariatric surgery are recommended. However, while FDA-approved weight loss drugs, such as lipase inhibitors (Orlistat; Xenical^®^), glucagon-like peptide-1 receptor agonists (GLP-1-RA) (Liraglutide and Semaglutide; Victoza^®^ and Ozempic^®^), and other appetite suppressants (Phentermine-topimarate; Qsymia^®^), improve weight loss [29,30,31], they are contraindicated during pregnancy and require a long washout period (≥2 months). Overall, more research is needed to fully elucidate the risk of short- and long-term adverse outcomes following prenatal drug exposure in offspring [32,33,34]. Bariatric surgery clearly improves weight loss; however, it also increases the risk of vitamin and micronutrient deficiencies, fetal growth restrictions (i.e., SGA offspring), maternal anemia, altered glucose homeostasis, and surgical complications [35,36,37,38]. In order to mitigate the risks, a 1–2 year waiting period is generally recommended prior to conception following bariatric surgery [36]. Clearly, the benefit–risk balance of pharmacological and surgical approaches must be carefully considered prior to implementation in overweight and obese couples attempting pregnancy. Thus, comprehensive lifestyle modification is the first-in-line treatment for weight loss in preparation for both spontaneous pregnancy and assisted reproductive technology (ART), with the added benefit that it may be safely continued throughout pregnancy for healthy maternal bodyweight gain, shortened labor, and reduced risk of gestational diabetes, hypertension, and Cesarian section [39,40,41]. Obviously, such programs must be carefully designed to avoid excess caloric restriction, ‘fad diets,’ and overly vigorous exercise, which may contribute to anovulation in some women [42], especially when added to the stress, anxiety, and depression associated with infertility [43].

An adjunctive approach in lifestyle modification programs may include the use of multi-ingredient nutrient supplements. Vitamins, antioxidants (AOs), and botanicals (e.g., phytonutrients) have gained popularity over the last decades because of their perceived health benefits and minimal side effects. Although scientific and regulatory challenges remain regarding safety, quality, and efficacy [44], select supplements appear to have scientific merit and may be devoid of major side effects whilst keeping within safe dosing ranges [45]. There is moderate-quality evidence in support of using green tea, coffee (black or green), and forskolin for weight loss, mainly attributed to the thermogenic, lipolytic, and appetite-suppressant effects of caffeine and plant polyphenols (e.g., epigallocatechin (EGCG) and chlorogenic acid (CGA)) [45,46]. Other AOs have also been shown to provide synergistic weight loss, and antioxidant and/or anti-inflammatory benefits, including coenzyme Q10 (CoQ10), α-lipoic acid (α-LA), and vitamin E (α tocopherol) [47,48]. However, while the theoretical basis and preclinical evidence on the use phytonutrients and antioxidants for treating obesity or related conditions, such as NAFLD [49], T2DM [50], and infertility [51,52], are promising, the quality of evidence from RCTs is generally low-to-moderate in strength.

As per the recommendations by the World Health Organization (WHO), supplementation with specific minerals and vitamins, such as iron and folic acid, is highly recommended as a normal part of prenatal care [53,54,55]. Cochrane systematic reviews also suggest that AO intake by either sex may improve live birth rates and clinical pregnancies [15,56]. Furthermore, it is well-known that omega-3 polyunsaturated fatty acids (ω-3 PUFAs), particularly docosahexaenoic acid (DHA) and eicosapentaenoic acid (EPA), are associated with beneficial effects on fetal neurodevelopment and pregnancy outcomes [57]. Emerging evidence also suggests that the use of L-arginine and creatine monohydrate by either sex may be beneficial for fertility improvement and fetal development [51,58,59,60]. Thus, the intake of a combination of specific dietary supplements during preconception and perinatal periods may be both advisable and safe, with favorable outcomes on weight management, fertility, and pregnancy [61,62].

The concept of multi-ingredient supplementation (MIS) to improve body composition has been tested previously [63,64], and a majority of weight loss products on the market contain a combination of ingredients [45]. Our group recently demonstrated that the provision of a blend of seven antioxidants (vitamin E, alpha-lipoic acid, and coenzyme Q10) and phytonutrients (beet root, green tea, green coffee, and forskolin) induced significant fat loss and WAT browning independent of physical activity while preserving muscle mass in obese mice [65]. In the current study, we aimed to assess the efficacy of the same multi-ingredient supplement with the addition of folic acid, L-arginine, creatine monohydrate, and ω3 PUFAs (EPA and DHA) (‘Fertility Enhancer’, FE) on weight loss, WAT expansion, NAFLD, and fertility in WD-fed C57BL/6J mice. Given that reproduction is a ‘couple concept,’ our primary aim was to determine the combined effects of weight loss and fertility enhancement in both sexes on overall reproductive success.

## 2. Methods

### 2.1. Ethics Approval

This study was approved by McMaster University Animal Research Ethics Board on June the 10th, 2019, and conformed to the standards of the Canadian Council on Animal Care (protocol code 19-117 AUP 16-05-15).

### 2.2. Animals, Housing, Food Consumption and Body Weights

All animals were housed in standard microisolator cages (12-h light/dark cycle at 22 °C) and provided food and water *ad libitum.* Health assessments were done daily, and bodyweights (BW) and food intake were measured twice weekly at the same hour in the morning using a calibrated scale.

### 2.3. Study Design

**Figure 1:**
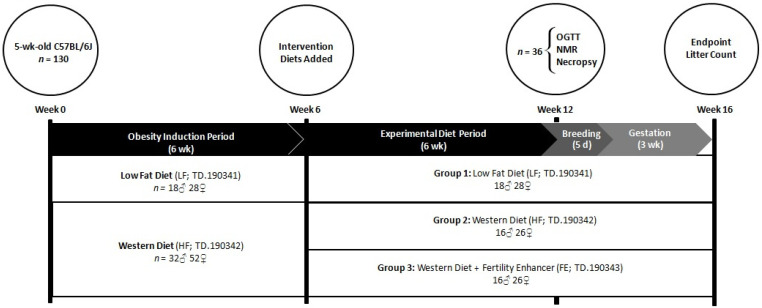
Study timeline for assessing the effects of the ‘Fertility Enhancer’ supplement on bodyweight, WAT expansion, NAFLD, and fertility in WD-fed C57BL/6J mice.

Five-month-old male (M) and female (F) C57BL/6J mice (JAX Laboratories, Bar Harbor, MA, USA) were fed a low-fat diet (LF, 3.64 kcal/g; *n* = 18 M, 28 F) or a high fat/sucrose WD (HF, 4.56 kcal/g; *n* = 32 M, 52 F) for six weeks, followed by six weeks of LF (*n* = 18 M, 28 F), HF (*n* = 16 M, 26 F), or HF combined with MIS (‘Fertility Enhancer’, FE, 4.50 kcal/g; *n* = 16 M, 26 F). At the six-week timepoint, the mice were matched by bodyweight and grouped into LF, HF, or FE conditions. At 12 weeks, a sub-set of animals (*n* = 6 per group and sex) underwent an oral glucose tolerance test and a body composition scan prior to being sacrificed for WAT, NAFLD, and gonadal pathology analyses. The remainder were harem-mated in a 1:2 male-to-female ratio for five days (e.g., one breeding/estrous cycle), and were, thereafter, singly housed during a three-week gestational period. Because C57BL/6J mice routinely lose 30% of their litters to cannibalism, PND 4 live litter size and survival were not obtained. All animals, including pups, were euthanized immediately upon confirmation of the litter (Figure 1).

### 2.4. Diets

All diets were manufactured by ENVIGO (Indianapolis, IN, USA) and supplements were supplied by Infinit Nutrition (Windsor, ON, Canada). The low-fat control diet (LF; TD.190341) contained 3.64 kcal/g made up of protein 19% kcal, carbohydrates 68.2% kcal (sucrose 120 g/kg), and fat 12.8% kcal. The high fat/sucrose Western diet (HF; TD.190341) contained 4.56 kcal/g made up by protein 15.3% kcal, carbohydrates 42.8% kcal (sucrose 345 g/kg), and fat 41.9% kcal. The Fertility Enhancer diet (FE; 190341) contained 4.5 kcal/g made up of protein 16.6% kcal, carbohydrates 37.1% kcal (sucrose 345 g/kg), and fat 46.2% kcal. FE also contained phytonutrients (green coffee bean extract; 2.5 g/kg, green tea extract; 0.75 g/kg and forskolin; 0.125 g/kg) and antioxidants (α-lipoic acid; 1 g/kg, CoQ10; 2.5 g/kg, vitamin E/α tocopheryl acetate; 2.188 g/kg and beetroot extract; 10 g/kg) for weight loss, as well as a blend of supplements for enhanced fertility, including L-arginine (13.66 g/kg), creatine monohydrate (30 g/kg), iron/ferric citrate (1.21 g/kg), folic acid (0.005 g/kg) and omega-3 (NutraSea; 18.12 g/kg).

### 2.5. Oral Glucose Tolerance Test (OGTT)

An oral glucose tolerance test (OGTT) was administered to a subset of animals from each experimental group at 12 weeks (*n* = 6 per group and sex). Briefly, the mice were fasted for six hours, gavaged with a 20% *w*/*v* β-D-glucose solution (2 g glucose/kg body weight) (Sigma-Aldrich, St. Louis, MO, USA), and bled via tail vein at 0 (baseline), 15, 30, 60, 90, and 120 min after glucose ingestion. Blood glucose levels were measured using a OneTouch Ultra Mini glucometer (LifeScan Canada ULC, Wayne, PA, USA) and area under the curve (AUC) was calculated according to the trapezoidal rule [66].

### 2.6. Time-Domain NMR Whole-Body Composition

The same subset of animals underwent in vivo body composition analysis using a time-domain NMR whole-body composition analyzer (Minispec LF90II, Bruker; Billerica, MA, USA). As previously described, NMR is a highly reproducible and accurate method of assessing body composition in obese mice [67]. Specifically, total fat mass, % bodyfat, and the Body Composition Index (BCI; total lean mass/total fat mass) were analyzed.

### 2.7. Necropsy

The mice were then sacrificed by cervical dislocation under isoflurane anesthesia. Testicles, ovaries, livers, and WAT (perigonadal, perirenal, mesenteric, and inguinal depots) were excised, weighed, and processed for histology, or were snap-frozen in liquid nitrogen and stored at −80 °C for downstream analyses.

### 2.8. Paraffin Embedding

Ovaries (*n* = 3 per group), testes (*n* = 6 per group), and livers (*n* = 8 per group; males *n* = 4 and females *n* = 4) were fixed in 10% formalin at room temperature for 24 h. After 24 h, cassettes were washed with running tap water and 50% ethanol, and stored in 70% ethanol until being paraffin embedded, sectioned (5-µm), and stained with haematoxylin & eosin (H&E) by the John Mayberry Histology Facility at McMaster University Medical Center.

### 2.9. Liver Pathology

H&E-stained liver sections were viewed under 200× total magnification with a Nikon 90i microscope, and one representative image was captured per animal. The percentage lipid droplet area per total area analyzed was then calculated using the ImageJ software (NIH, Bethesda, MD, USA). Specifically, lipid droplet area was measured using a color threshold and any measured artifact or unmeasured lipid droplets were manually accounted for. A subset of livers from each group was also stained by Oil O Red and TUNEL methods for confirmation of NAFLD and general pathology. The criteria used for classifying NAFLD were hepatosteatosis (grade 1), upregulation of inflammation, apoptosis and senescence (grade 2), and liver damage/fibrosis (grade 3–4).

### 2.10. Ovarian Pathology

Ovarian pathology was assessed on four consecutive 5-µm paraffin cross-sections per animal following haematoxylin and eosin staining. H&E sections were imaged using a Nikon 90i microscope at 200× total magnification and analyzed using ImageJ software (NIH, Bethesda, MD, USA).

Follicles were classified as primordial if they featured an oocyte surrounded by up to a single layer of squamous granulosa cells. If the granulosa cells were predominantly cuboidal, less than two complete layers was indicative of a primary follicle while two or more layers with no visible antral spaces indicated a secondary follicle. Once the antral cavity had begun to form, a few small fluid-filled spaces between granulosa cells indicated an early antral follicle while the presence of one large antral cavity was characteristic of an antral follicle. If a follicle appeared to be healthy but no oocyte was visible in the section, the follicle was deemed unclassifiable healthy. If a follicle showed any signs of degeneration, irrespective of any other identifying features, it was classified as atretic. Evidence of atresia included deformed shape, vacuolation, loss of the nuclear membrane, fragmentation of the oocyte, and disorganized or pyknotic granulosa cells. Although they do not contain an oocyte, corpora lutea were also counted in each ovarian section. These structures were identified as large masses of granulosa cells with increased cytoplasmic-to-nuclear ratios.

Once all follicles were counted in each ovarian section individually, the four sections of each animal were cross-compared to ensure each unique follicle was counted only once per ovary. For all follicle types, counts per section were then normalized to the section area while counts per ovary were normalized to the total volume of the four sections (total volume = area of cross section x section thickness x number of sections).

### 2.11. Testicular Pathology

Testicular pathology was assessed on 5-µm paraffin cross-sections following haematoxylin and eosin staining. Sections were imaged using a Nikon 90i microscope at 200× total magnification and from three to eight representative images were captured per animal, depending on the quality of the section.

A grading scale was established to rate the visible pathology of the testicular seminiferous tubule cross sections. Tubules were assigned a score of 0 if the seminiferous epithelium appeared healthy and organized and cells appeared tightly packed. If the cell–cell adhesions were starting to loosen, the tubule was given a score of 1 and, if this loosening was paired with visible disruptions in the epithelium, the tubule was assigned a score of 2. Tubules that were visibly disorganized with significant epithelial disruption received a score of 3. Values of 0.5, 1.5, and 2.5 were also assigned if the pathology of a tubule appeared to fall in between classifications. The diameters of the roundest seminiferous tubule cross-sections in each representative image were also measured using ImageJ software (NIH, Bethesda, MD, USA).

#### Terminal Deoxynucleotidyl Transferase (TdT)-Mediated dUTP Nick-End Labeling (TUNEL)

Apoptotic nuclei in gonadal tissues were assessed by TUNEL staining of paraffin sections as per manufacturer’s instructions using Abcam assay kit (ab206386). In brief, Terminal deoxynucleotidyl Transferase (TdT) was added to the tissue sections and allowed to bind with any exposed 3′-OH DNA fragment ends that had been generated by apoptosis. The TdT catalyzed the addition of biotin-labeled deoxynucleotides to these fragments, which were then detected by a streptavidin-horseradish peroxidase (HRP) conjugate. Next, diaminobenzidine was applied and reacted with the HRP, producing an insoluble brown substrate at each DNA fragmentation site. Counterstaining was done with methyl green to facilitate quantification. TUNEL-stained ovarian tissues were analyzed via light microscopy at 200× total magnification (Nikon 90i). Apoptosis was quantified by counting the number of TUNEL-positive granulosa cells per follicle and per ovarian section. All visible follicles were then counted and classified as small, medium, or large to roughly reflect pre-antral, early antral, and antral follicles, respectively. Similarly, TUNEL-stained testicular tissues were analyzed at 200× magnification and TUNEL-positive germ cells were counted. The number of apoptotic nuclei per seminiferous tubule cross-section were counted for 50 tubules in each testicular section.

### 2.12. RNA Isolation

Briefly, snap-frozen WAT, liver, ovary, and testicles were pulverized with a Cellcrusher Tissue Pulverizer (Cellcrusher, Portland, OR, USA) on dry ice. Total RNA was extracted with a tissue-specific RNeasy Mini Kit (Qiagen, Germantown, MD, USA; Cat No. 74104 and 74804, respectively), eluted with RNase-free water, and checked for OD_260_/OD_280_ purity using a Nanodrop 1000 Spectrophotometer (Thermo Fisher Scientific, Waltham, MA, USA). Stock RNA was stored at −80 °C.

### 2.13. cDNA Synthesis

Reverse transcription was performed on 1 μg (WAT and liver), 0.25–1 μg (ovary), and 1–2 μg (testicle) of RNA using a SuperScript™ IV VILO™ Master Mix (Thermo Fisher Scientific; Waltham, MA, USA; Cat No. 11756500) per the manufacturer’s protocol. The reaction mixtures were incubated in a T100 Thermal Cycler (Bio-Rad, Hercules, CA, USA) with thermocycling conditions of 25 °C for 10 min (Annealing), 50 °C for 10 min (Elongation), and 85 °C for 5 min (Enzyme Inactivation). Synthesized cDNA was diluted with nuclease-free water to obtain a concentration of 5.5 ng/µL and then stored at −80 °C.

### 2.14. Quantitative RT-PCR

Gene expression was determined by running 4.5 µL of cDNA template (5.5 ng/ µL) to a final volume of 10 µL on a CFX384 Real-Time System (Bio-Rad, Hercules, CA, USA) using a TaqMan Fast Advanced Master Mix (Thermo Fisher Scientific; Waltham, MA, USA; Cat No. 4444557). Taqman cycling conditions were: 20 s at 95 °C for initial denaturation, followed by 40 cycles with 3 s denaturation at 95 °C and 30 s annealing and elongation at 60 °C. The fluorescence threshold was automatically set above the background level. All samples were run in triplicate, including a no-template control. Fold-increase in mRNA was calculated by the $2^{-\Delta \Delta C_{T}}$ method and expression levels were normalized to tissue-appropriate reference genes β_2_ microglobulin (β2M) or peptidylprolyl isomerase A (PPIA). All probes were purchased from Thermo Fisher Scientific.

### 2.15. Perigonadal WAT Immunoblotting

MP Biomedicals FastPrep-24 Classic Instrument was used to homogenize perigonadal WAT for Western blotting per the manufacturer’s instructions. Briefly, 50–60 mg of tissue was obtained per animal and combined with 2 µL Halt Protease and Phosphatase Inhibitor (Thermo Fisher Scientific, 78440) and 198 µL Pierce RIPA Buffer (Thermo Fisher Scientific, 89901). Tubes were then spun in the FastPrep-24 for 45 s at 6m/s and placed on ice to cool for 60 s. These spin steps were repeated twice more, and the resulting homogenates were kept on ice for one hour. Finally, all samples were centrifuged three times for 15 min each at 12,000 g and 4 °C. Only the supernatant was retained between spins while the pellet and lipid layer were discarded.

Thermo Fisher Scientific Pierce BCA Protein Assay Kit (23225) was used to determine protein concentration of homogenates according to the manufacturer’s instructions. Samples were then denatured in 6× Laemmli buffer (Bio-Rad, 161-0747), in ratios that allowed for equal protein and volume loading of gels, and heated at 95 °C for 5 min. A total of 25 µg of protein was added to each well of the 4–20% Criterion TGX Precast Midi Protein Gel (Bio-Rad, 5671094) and electrophoresis was applied at 70 V for 15 min, followed by 120 V for 1 h and 20 min. Semi-dry transfers onto 0.2 µM nitrocellulose membranes (Bio-Rad, 1704159) were then performed using the Bio Rad Trans-Blot Turbo Transfer System at 2.5 A for 10 min.

Membranes were then developed in PonceauS solution (Sigma-Aldrich, P7170-1L) and imaged using Bio Rad ChemiDoc MP Imaging System before being blocked for one hour at room temperature in 5% bovine serum albumin (Sigma-Aldrich, A9647) in tris-buffered saline (Bio Rad, 1706435) and 0.01% TWEEN20 (Sigma-Aldrich, P2287). Membranes were then left to incubate in primary antibody solution (1:500–1000 dilution in 5% BSA or 1% NFDM) overnight at 4 °C under gentle agitation. The following day, membranes were washed with 1x TBST (tris-buffered saline with 0.01% TWEEN20) and incubated in anti-rabbit or anti-mouse secondary antibody (1:10,000 dilution in 5% BSA) for one hour at room temperature under gentle agitation. Following additional washes in 1x TBST, membranes were incubated for 5 min in Clarity Western ECL Substrate (Bio Rad, 170-5061), prepared as per the manufacturer’s instructions, and imaged using Bio Rad ChemiDoc MP Imaging System. Optical densities of protein bands were quantified using ImageJ software after subtracting background and normalizing to Ponceau S staining.

### 2.16. Statistical Analyses

For omnibus F-tests, 2 × 3 factorial ANOVA (biological sex x diet), 2 × 3 repeated measures ANOVA, or one-way ANOVA test were used, followed by Fisher’s LSD post hoc testing to specify group differences (Statistica v. 12, Statsoft Inc.). Specifically, one-way ANOVAs were used for all reproductive outcomes, gonadal pathology, and gonadal mRNA levels, while obesity outcomes (e.g., bodyweights, WAT, and NAFLD) were analyzed by factorial ANOVAs. Priority comparisons were: 1. HF vs. LF for confirmation of obesity and associated comorbidities, and 2. FE vs. HF to assess the efficacy of the ‘Fertility Enhancer’. Statistically significant differences between priority groups are denoted in tables and figures with symbols (*p* ≤ 0.05). All raw data and statistical analyses are available upon reasonable request.

## 3. Results

### 3.1. Food Intakes, Body Weights, and Body Conditions

**Figure 2:**
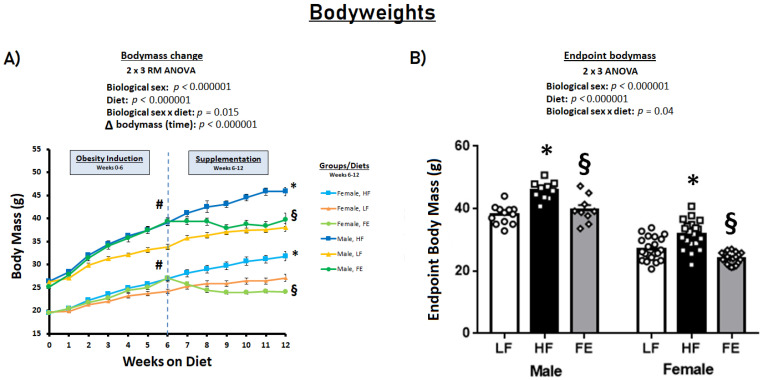
Effects of the ‘Fertility Enhancer’ supplement on bodyweights in Western diet-fed C57BL/6J mice. (**A**) Longitudinal changes in body mass. (**B**) Endpoint body mass. * Significantly different from LF (*p* ≤ 0.05). § Significantly different from HF (*p* ≤ 0.05). # Significantly different from baseline within groups.

Six weeks of obesity-induction by a Western diet resulted in significantly higher energy intake and pronounced body weight gain vs. the LF control diet in C57BL/6J mice (*p* < 0.001) (Figure 2A). During the subsequent six-week supplementation period, energy intake continued to be higher in HF vs. LF animals (*p* < 0.05) (Table 1), with endpoint bodyweights that were significantly higher in the HF groups (*p* < 0.001) (Figure 2B). Conversely, the FE-treated mice did not gain weight during the supplementation period, with bodyweights either normalized (males) or marginally lower (females) than LF at endpoint (Figure 2B).

By design, the observed weight loss in this study was partially driven by central effects of specific ingredients in the FE diet, such as alpha lipoic acid [68], as indicated by a significantly lower energy intake in the FE group. To further explore this concept, we conducted a pair-feeding experiment in a similar murine model and found that up to 30–40% of weight loss may be attributed to appetite suppression (Supplementary Figure S1). The mice in the current study were carefully monitored for body condition and signs of stress, and there were no instances of lethargy or emaciation. Barbering and hair loss were not different between groups and occurred in five animals across groups over the four-month study period.

### 3.2. Blood Glucose

**Figure 3:**
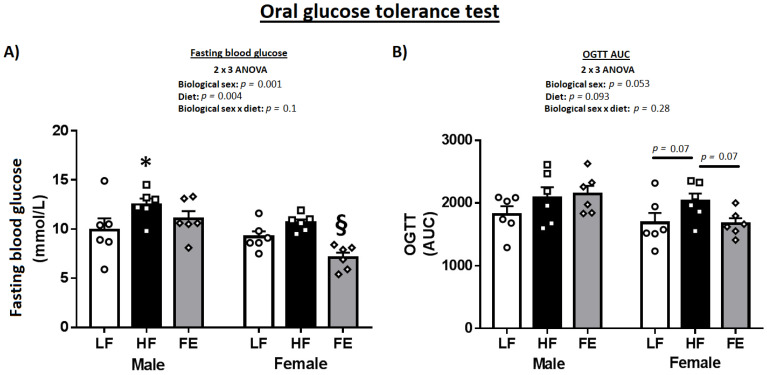
Effects of the ‘Fertility Enhancer’ supplement on fasting blood glucose and oral glucose tolerance test in Western diet-fed C57BL/6J mice. (**A**) Fasting blood glucose. (**B**) Area under the curve (AUC) from oral glucose tolerance test (OGTT) * Significantly different from LF (*p* ≤ 0.05). § Significantly different from HF (*p* ≤ 0.05).

The OGTT indicated more pronounced benefits of the ‘Fertility Enhancer’ in females vs. males (Table 1 and Figure 3A,B). Specifically, fasting blood glucose and AUC were significantly lower in FE-treated vs. HF females (*p* < 0.05), while only fasting blood glucose was marginally affected by supplementation in males.

### 3.3. Body Composition, WAT Expansion, and WAT Biochemistry

**Figure 4:**
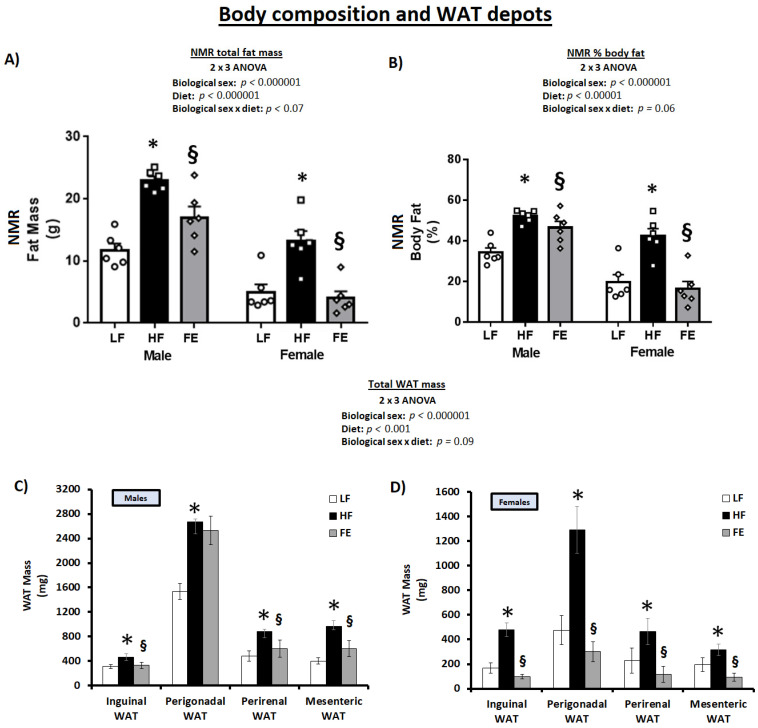
Effects of the ‘Fertility Enhancer’ supplement on total fat mass, % body fat, and WAT depots in Western diet-fed C57BL/6J mice. (**A**) Total fat mass from NMR. (**B**) Percentage body fat from NMR. (**C**) White adipose tissue mass in males. (**D**) White adipose tissue mass in females. * Significantly different from LF (*p* ≤ 0.05). § Significantly different from HF (*p* ≤ 0.05).

Total body fat and % bodyfat were significantly higher in HF vs. LF at endpoint (Figure 4A,B) (*p* < 0.05), reflective of an expansion of all WAT depots (Figure 4C,D) (*p* < 0.05). Total body fat, % body fat, and WAT expansion were significantly lower in male FE vs. HF mice and normalized to LF levels in FE-treated females. In addition, the ‘Body Composition Index’ was higher in FE vs. HF animals, indicative of an overall improved muscle-to-fat ratio and body composition across sexes (Table 1, *p* < 0.05).

Collectively, the in vivo NMR scans, necropsy data, and OGTT test strongly suggest that the ‘Fertility Enhancer’ induced weight loss, lowered WAT mass, improved body composition, and mitigated hyperglycemia in WD-fed mice, with benefits that were potentially more pronounced in females vs. males.

### 3.4. WAT PGC-1α mRNA Levels and Total OXPHOS Expression

We previously demonstrated that the same blend of antioxidants (vitamin E, alpha-lipoic acid, coenzyme Q10) and plant extracts (green coffee bean, green tea, mint, and beet root) used herein induced significant fat loss and WAT browning in male DIO mice [65].

**Figure 5:**
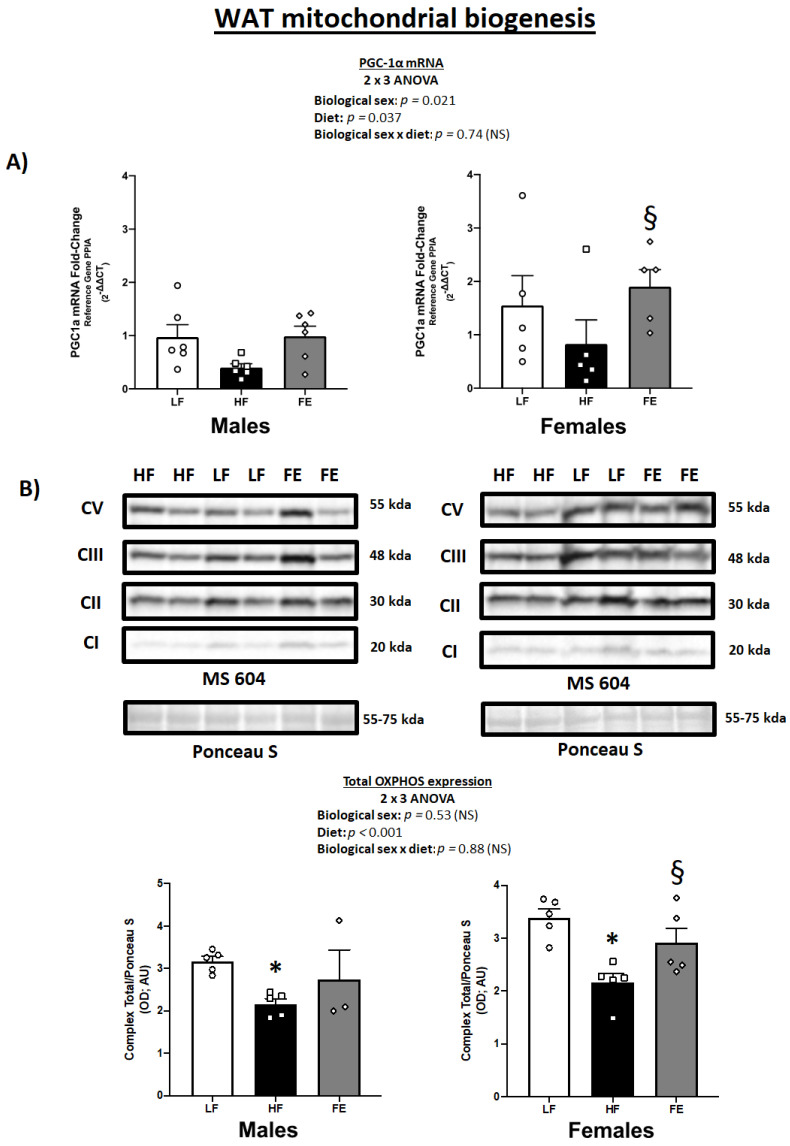
Effects of the ‘Fertility Enhancer’ supplement on PGC-1α levels and OXPHOS protein expression in perigonadal WAT in Western diet-fed C57BL/6J mice. (**A**) PGC1α mRNA levels in males and females. (**B**) OXPHOS protein expression in males and females. * Significantly different from LF (*p* ≤ 0.05). § Significantly different from HF (*p* ≤ 0.05).

As expected, we found a main effect of diet on PGC-1α mRNA levels in perigonadal WAT in the current study (*p* = 0.037) (Figure 5A), with generally higher PGC-1α levels in FE-treated vs. HF animals across both sexes. While this only reached statistical significance in females, the overall data pattern suggests that chronic high fat/sucrose feeding causes PGC-1α downregulation, which is rescued by concurrent supplementation with the ‘Fertility Enhancer’.

Mitochondrial protein expression analyses confirmed that Western diet-fed animals exhibited lower total OXPHOS levels vs. LF and that this may be reversed by the ‘Fertility Enhancer’ supplement (Figure 5B) (*p* < 0.001). Individual complex expression indicated main effects of diet on CII (*p* = 0.006), CIII (*p* < 0.001), and CV (*p* = 0.027), as well as borderline significance for CI (*p* = 0.07), while CIV was not detected by immunoblotting in this study.

### 3.5. Liver Pathology

#### 3.5.1. Hepatosteatosis

**Figure 6:**
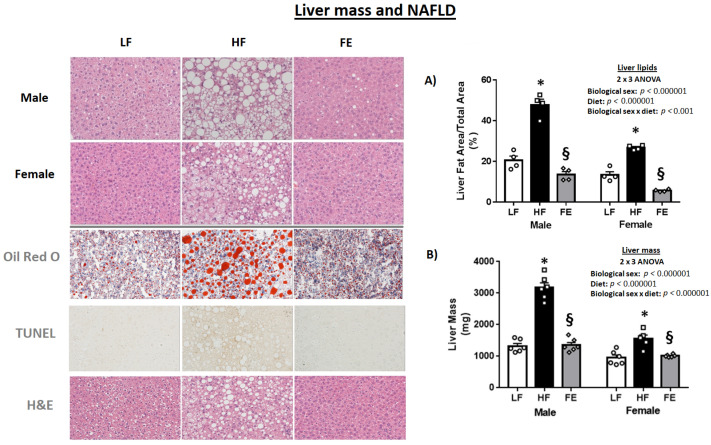
Effects of the ‘Fertility Enhancer’ supplement on NAFLD and liver mass in Western Diet-fed C57BL/6J mice. (**A**) Liver lipid droplet area. (**B**) Liver mass. * Significantly different from LF (*p* ≤ 0.05). § Significantly different from HF (*p* ≤ 0.05).

Following 12 weeks of high fat/high sucrose feeding, hepatosteatosis was pronounced and liver masses were significantly higher in HF vs. LF mice (*p* < 0.05) (Figure 6A,B), indicative of early state NAFLD. Remarkably, both male and female FE-treated mice exhibited significantly lower levels of liver lipids vs. the HF and LF groups, suggestive of the supplement having a potent lipolytic effect in the liver. Oil Red O and TUNEL stains largely confirmed pathology reversal with the ‘Fertility Enhancer’.

#### 3.5.2. Liver mRNA Levels

**Figure 7:**
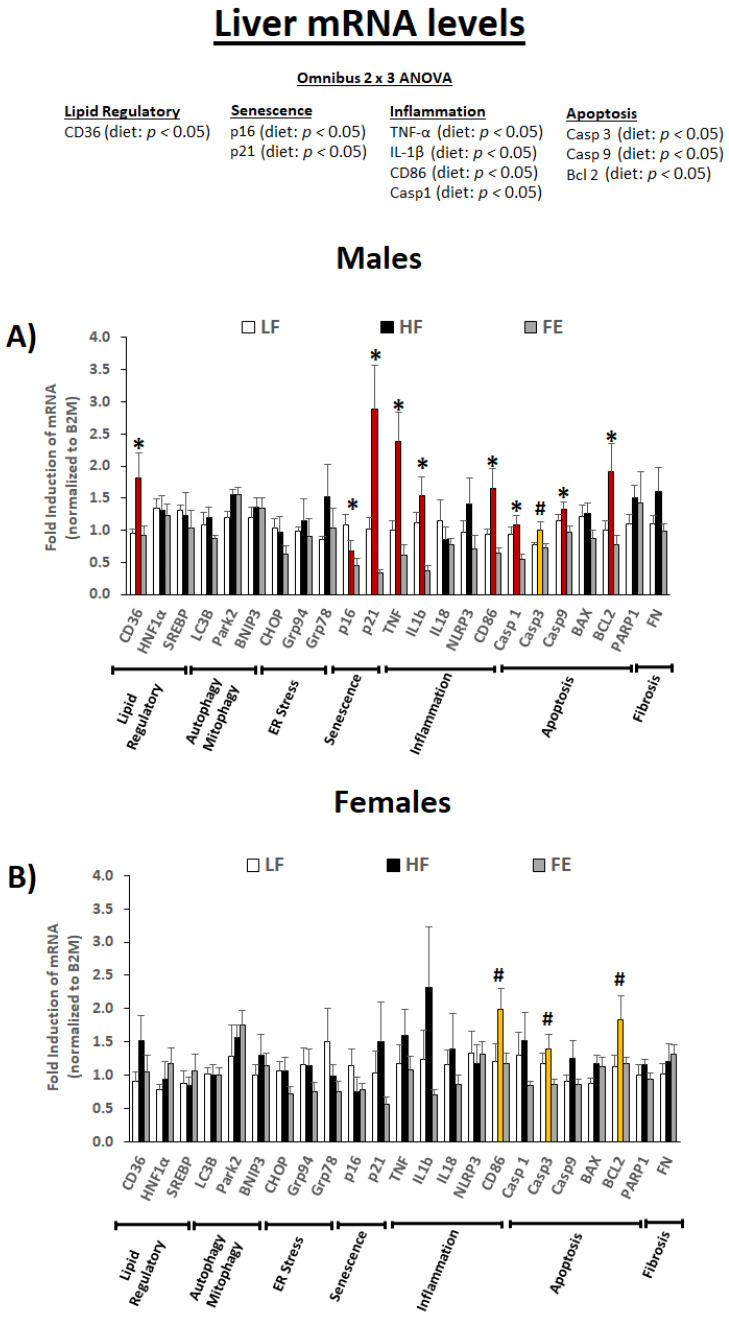
Effects of the ‘Fertility Enhancer’ supplement on liver mRNA levels in Western diet-fed C57BL/6J mice. * Significant one-way ANOVA within sex (*p* ≤ 0.05). # Borderline significant one-way ANOVA within sex (*p* ≤ 0.1).

We next assessed liver mRNA levels for genes involved in lipid regulation, senescence, inflammation, and apoptosis (Figure 7A,B). The omnibus 2 × 3 ANOVA revealed a main effect of diet type on CD36, p16, p21, TNF-α, IL-1β, CD86, CASP1, CASP3, CASP9, and BCL2 (*p* < 0.05).

Collectively, our data indicate that high fat/sucrose feeding induced hepatosteatosis, cell senescence, inflammation, and apoptosis in the liver (e.g., stage 1–2 NAFLD), and that these pathological changes were largely mitigated by the ‘Fertility Enhancer.’ While the protective benefits appear to be more pronounced in males vs. females on the transcriptional level, the overall data pattern and lipid clearance results indicate similar effects across sexes.

### 3.6. Mating Behavior and Reproductive Outcomes

**Figure 8:**
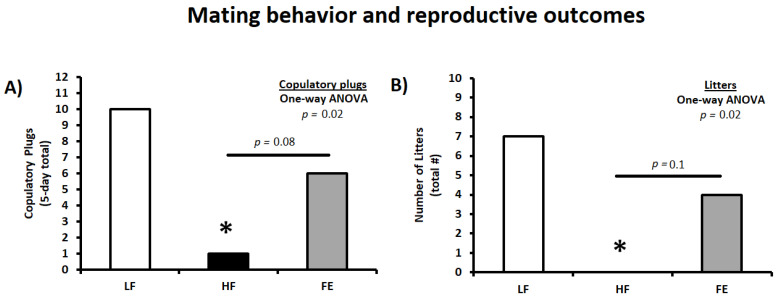
Effects of the ‘Fertility Enhancer’ supplement on mating behavior and reproductive outcomes in Western diet-fed C57BL/6J mice. (**A**) Five-day total copulatory plugs in females. (**B**) Total number of litters. LF = Low Fat Diet, HF = Western Diet (WD), and FE = ‘Fertility Enhancer’ multi-nutrient supplement with WD. * Significantly different from LF (*p* ≤ 0.05).

Following the obesity induction period, animals were harem-bred for five days to determine the effects of high fat/sucrose feeding and FE supplementation on mating behavior and reproductive success. Thus, vaginal plugs were counted daily during the mating period and indicated that the Western diet significantly impaired mating frequency in HF couples (*p* < 0.05 vs. LF) (Table 2 and Figure 8A,B). This is consistent with previous findings in C57BL/6J mice when using one five-day breeding/estrous cycle [69]. Obesity-induced infertility was partially mitigated by the ‘Fertility Enhancer,’ with copulatory plugs (six vs. zero, *p* = 0.08), number of litters (four vs. zero, *p* = 0.1), and reproductive success being higher in FE vs. HF couples (litters/plugs; 67% vs. 0%).

### 3.7. Gonadal Pathology

In addition to in vivo analyses of mating behavior and reproductive outcomes, we examined ovarian and testicular tissue pathology in a small subset of animals using standard histology and quantitative PCR techniques. Because of limited gonadal tissue, protein expression studies could not be done.

#### 3.7.1. H&E and TUNEL Staining

Generally, gonadal mass, seminiferous tubule diameter, and ovarian volume were heavier and larger, respectively, in HF vs. control animals, and the supplement appeared to normalize these pathological changes across both sexes (Table 3). Previous studies have reported similar changes to gonadal morphometry in obese animals [70], likely due to a massive intra-organ lipid accumulation [69]. In the current study, testicular pathology scores based on cell adhesion and organization of seminiferous epithelia in male mice were largely unremarkable. Additionally, while TUNEL-positive germ cells in seminiferous tubules were numerically higher in HF vs. LF and FE animals, the data were too variable to draw definite conclusions.

**Figure 9:**
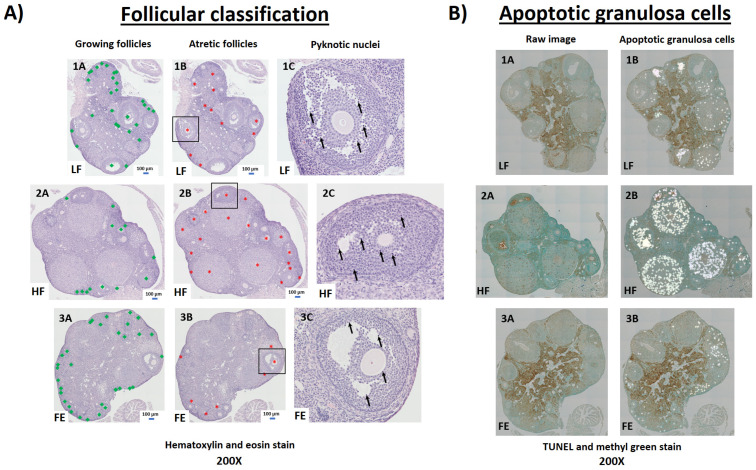
Ovarian follicular pathology by hematoxylin and eosin (H&E) and TUNEL staining. (**A**) Four consecutive, ovarian paraffin-sections (5 μm) per animal were stained with H&E and imaged via light microscopy for follicular pathology (200×). All follicles, including primary, secondary, early antral, and antral (e.g., excluding primordial follicles and corpus luteum) were counted and categorized as either growing (A; green squares) or atretic (B; red stars). Follicles containing dark, pyknotic nuclei within the granulosa cells were considered atretic (C; arrows). (**B**) One ovarian paraffin-section (5 μm) per animal was stained via terminal deoxynucleotidyl transferase-mediated dUTP nick-end labeling (TUNEL) and imaged via light microscopy to identify apoptotic granulosa cells (200×). LF = Low Fat Diet (Panel 1), HF = Western Diet (Panel 2), FE = Fertility Enhancer Diet (Panel 3).

**Figure 10:**
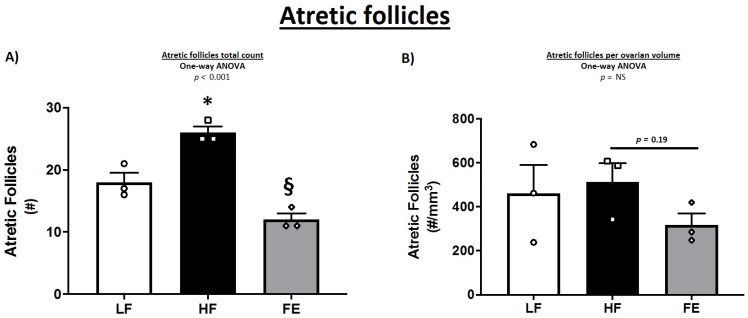
Effects of the ‘Fertility Enhancer’ supplement on follicular atresia in Western diet-fed C57BL/6J mice. (**A**) Total atretic follicles per section. (**B**) Total atretic follicles per ovarian volume. LF = Low Fat Diet, HF = Western Diet (WD), and FE = ‘Fertility Enhancer’ multi-nutrient supplement with WD. * Significantly different from LF (*p* ≤ 0.05). § Significantly different from HF (*p* ≤ 0.05).

In female mice, the classification and counting of ovarian follicles were indeed suggestive of a detrimental effect of the Western diet, with a lower number of healthy and growing follicles in HF vs. LF and FE groups (Table 3 and Figure 9A). These observations are consistent with previous findings in obese mice [70]. In line with this, WD-fed animals exhibited more atretic follicles per ovary compared to LF and FE-treated mice (*p* < 0.001) (Figure 9A and Figure 10A,B), which has been shown previously [69,71,72]. Lastly, TUNEL-positive granulosa cells were more abundant in follicles of HF vs. LF and FE mice (Figure 9B), while the sample size was too small for statistical significance.

#### 3.7.2. Gonadal mRNA Levels

**Figure 11:**
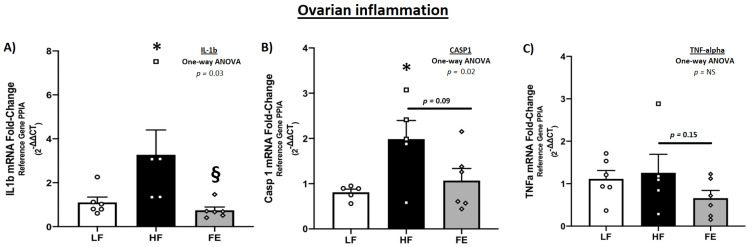
Effects of the ‘Fertility Enhancer’ supplement on key drivers of the inflammatory cascade in ovarian tissue of Western diet-fed C57BL/6J mice. (**A**) Ovarian IL-1β mRNA. (**B**) Ovarian CASP1 mRNA. (**C**). Ovarian TNF-α mRNA. LF = Low Fat Diet, HF = Western Diet (WD), and FE = ‘Fertility Enhancer’ multi-nutrient supplement with WD. * Significantly different from LF (*p* ≤ 0.05). § Significantly different from HF (*p* ≤ 0.05).

Messenger RNA levels of key inflammatory drivers were significantly higher in ovarian tissue of WD-fed vs. control mice (*p* < 0.05 IL-1β and Caspase 1) (Figure 11A–C), consistent with a chronic inflammatory state and follicular atresia following obesity induction. These observations are similar to those of Gao et al. [69], although TNF-α levels were not markedly elevated in the current study. Furthermore, IL-1β, CASP1 and TNF-α were lower in FE-treated vs. HF animals, which is suggestive of an anti-inflammatory effect of the supplement. Markers of mitochondria (ND1 and SDHA), cell cycle inhibition (p21), and apoptosis (CASP3 and CASP9) were not significantly different between treatments (Table S1).

In testicular tissue, messenger RNAs regulating inflammation (CASP1, TNF-α, IL-1β, IL-6, CD86, and NLR3P), apoptosis (CASP3 and CASP9), DNA repair (PARP1), and androgen receptor (AR) were generally higher in the testicles of both HF- and FE-treated animals vs. controls (Table S1). In other words, while the ‘Fertility Enhancer’ normalized certain aspects of obesity-associated testicular pathology (Table 3), the transcriptional data were largely unremarkable.

Because of limited gonadal tissue for both males and females, protein expression analyses were not done in this study, which may have added more insight into underlying mechanisms.

## 4. Discussion

Overweight and obese couples are incentivized more than ever to seek alternative approaches to preconception weight loss and fertility enhancement, including the use of various dietary supplements in their daily nutritional regimen. In this pre-clinical study, the findings indeed suggest that daily consumption of a combined weight loss and fertility-enhancing, multi-ingredient supplement (‘Fertility Enhancer’, FE), consisting of a blend of antioxidants (alpha-lipoic acid, co-enzyme Q10, and vitamin E), phytonutrients (green tea, green coffee, and forskolin), vitamins (folic acid), amino acids (L-arginine), ω3 PUFAs (EPA and DHA), and creatine monohydrate, may be beneficial for weight loss and fertility enhancement in overweight and obese couples. Specifically, we report that the FE supplement mitigated body weight gain, white adipose tissue (WAT) expansion, and stage 1–2 NAFLD in both male and female Western diet-fed mice, and increased mating frequency and number of litters over WD-fed controls, at least partially by attenuating ovarian inflammation and atresia. In line with the ‘couple concept’ [14], our primary aim was to explore the combined effects of weight loss and fertility treatment across the sexes; thus, not to delineate the independent effects of weight loss vs. fertility enhancement or the unique contributions of male vs. female factors to the observed reproductive benefits.

We have previously demonstrated that an almost identical multi-ingredient supplement effectively mitigated bodyweight gain and WAT expansion in male DIO mice fed a very high-fat diet (60% of total kcals) [65]. Herein, we extend these findings to both male and female mice on a Western diet (42% of total kcals and 345 g/kg sucrose) and demonstrate potent lipolytic effects in the liver with an attenuation of cell senescence, inflammation, and apoptosis (stage 1–2 NAFLD). While neither study was designed to assess the independent effects of each ingredient, it is likely that weight loss was driven by multiple different pathways, which is the impetus of using a blend of ingredients with complimentary modes of action. Collectively, our preclinical studies indicate that both central and peripheral factors, including appetite suppression, enhanced lipolysis, induction of WAT browning, and anti-inflammatory effects, drive the observed weight loss benefits (current study and [65]).

In line with this, moderate-quality evidence from human RCTs suggests that green tea [73,74] and coffee extracts (black and green) [75,76] induce weight loss due to the thermogenic, lipolytic, and appetite suppressant effects of caffeine, EGCG and CGA [45,46]. Furthermore, polyphenols exhibit antioxidant (AO) properties that may mitigate the generation of reactive oxygen species (ROS) induced by high fat/sucrose diets and energy surplus, alleviating insulin resistance, oxidative damage, and inflammation in insulin-sensitive tissues [48]. Some evidence also suggests that polyphenols may protect against NAFLD, which is possibly linked to the antioxidant response [77]. Other types of AOs, such as coenzyme Q10 (CoQ10), α-lipoic acid (α-LA), and vitamin E (α tocopherol), may provide synergistic weight loss, antioxidant, and anti-inflammatory benefits [47,48]. Recent meta-analyses have concluded that CoQ10 supplementation alleviates lipid peroxidation and inflammation in conditions of metabolic syndrome [78], and lowers total cholesterol and low-density lipoprotein (LDL) levels in diabetic patients [79]. Bobe and colleagues reported that α-LA supplementation reduced body mass index and urinary F2-isoprostanes (marker of lipid peroxidation) in overweight and obese women [80], and recent meta analyses generally confirm a modest but significant weight loss following α-LA supplementation [81,82,83]. These benefits may be attributed to antioxidant effects and/or central, appetite suppressing signaling (e.g., hypothalamic AMPK), as demonstrated in both preclinical models and in humans [68,84]. Vitamin E (α tocopherol) is a lipid soluble vitamin with antioxidant properties that has been shown to mitigate features of metabolic syndrome, NAFLD, oxidative damage, and inflammatory markers in both animal and human studies [77,85,86]. Forskolin is a mint extract derived from the roots of *Coleus forskohlii*, which is commonly used for weight loss [46]. Godard et al. demonstrated that forskolin supplementation resulted in significant loss of fat mass in overweight and obese individuals [87], and other double-blind RCTs have reported insulin-lowering effects [88]. Collectively, there is low-to-moderately strong RCT evidence in support of certain plant extracts, antioxidants, and vitamins in weight management. A similar supplementation strategy may also work for treating NAFLD considering its shared etiological origins with obesity. Thus, research-proven supplements may be viable alternatives to pharmaceutical and surgical approaches for preconception weight loss and/or healthy weight gain during pregnancy. Although multi-ingredient supplementation has yet to be evaluated in this context, caffeine (≤200–300 mg/day), antioxidants, and prenatal micronutrients (vitamins and minerals) are generally regarded as safe during pregnancy [61,62].

While pre-conception weight loss by itself is beneficial and leads to increased pregnancy rates and live births [89,90], specific supplements have independent benefits on female fertility and are recommended as a normal part of prenatal care. For example, iron and folic acid, alone or as parts of a multivitamin tablet or gummy, are recommended before conception and during pregnancy to prevent maternal anemia and birth defects [53,54,55]. Supplementation with other vitamins, such as vitamins C, D, and E, is not essential for prenatal care but is considered safe [91,92,93]. Evidence also suggests that the provision of extra ω-3 PUFAs, particularly docosahexaenoic acid (DHA) and eicosapentaenoic acid (EPA), is associated with longer pregnancies, higher birth weights, reduced incidence of preterm birth, and beneficial effects on fetal neurodevelopment [57]. Although the quality of evidence evaluating antioxidant treatment on female subfertility is generally low [15], a recent Cochrane review indicates that AOs may improve live birth rates and clinical pregnancies [62]. Supplementation of antioxidant vitamins with L-arginine appears to protect against preeclampsia [94], while arginine alone may benefit fetal development [60]. A retrospective study in a cohort of 287 pregnant women found that maternal excreted levels of creatine are associated with fetal growth [58], suggestive of a beneficial effect of prenatal creatine supplementation. Because the benefits and safety of enhanced creatine intake in healthy women are well-documented [95], and preclinical data suggest that it may protect against certain pregnancy complications [59], future studies in low-risk pregnant women are certainly warranted [96].

As discussed previously, fertility is a ‘couple concept,’ and male factors account for one third of all infertility cases. Thus, dietary supplements for male reproductive health are a rapidly growing market; however, only 10% and 20% of products for male fertility and testosterone/erectile dysfunction, respectively, are considered to be of high efficacy based on dosage and active ingredients [51,97]. In a recent Cochrane review, Smits and colleagues found low-quality evidence in support of AOs for male fertility enhancement [15,56]. Nutraceuticals with clinical evidence for improved sperm parameters based on RCTs, meta-analyses, and systematic reviews include folic acid, L-arginine, α-lipoic acid, α tocopherol, coenzyme Q10, and EPA/DHA (for a comprehensive list and minimal effective dosages see Garolla et al. [51]). Because mitochondria play a major role in sperm motility, and spermatozoa are particularly vulnerable to oxidative stress generated by mitochondrial complexes I and III, nutraceuticals with antioxidant capacity or other mitochondrial benefits are of major interest [98]. While α-lipoic acid, α tocopherol, and coenzyme Q10 primarily act as ROS scavengers, EPA/DHA, creatine, and L-arginine may improve mitochondrial function and sperm motility by affecting phospholipidome, ATP levels, and nitric oxide availability (e.g., eNOS), respectively [99,100,101]. In terms of nutraceuticals for testosterone and erectile dysfunction, L-arginine, *Eurycoma longifolia*, *Tribulus terrestris*, Horny goat weed, *Panax ginseng*, and Yohimbine may have some scientific merit [97], although safety concerns have been raised in some cases.

Obesity predisposes both sexes to infertility, reduces the efficacy of fertility treatments, and increases the risk of birth defects. Affected couples experience substantial emotional, physical, and financial distress [12], and the development of cost-effective, adjunctive therapies to traditional weight management; therefore, fertility treatments is merited. Because obesity and fertility may be considered ‘couple concepts’ [14,102], interventions that can be applied universally or easily modified to target each sex are highly desirable. Because of an increased popularity and ongoing regulatory concerns related to dietary supplements for weight loss and fertility, we therefore advocate for more preclinical and clinical data related to safety, quality, and efficacy.

In this preclinical study, we aimed to assess the efficacy of the daily intake of a blend of antioxidants (alpha-lipoic acid, co-enzyme Q10, and vitamin E) and plant extracts (green tea, green coffee, forskolin, and beetroot), combined with specific fertility-enhancing agents (folic acid, iron, L-arginine, and creatine monohydrate) (‘Fertility Enhancer; FE), to improve weight loss, early stage non-alcoholic fatty liver disease (NAFLD), and fertility in male and female Western diet-fed C57BL/6J mice. We found that multi-ingredient supplementation effectively mitigated bodyweight gain, white adipose tissue expansion, dysglycemia, and stage 1–2 NAFLD in WD-fed mice, which is consistent with our previous observations in a more severe diet-induced obesity model [65]. Importantly, the ‘Fertility Enhancer’ also improved reproductive success in obesity, at least in part by attenuating ovarian inflammation and atresia. We conclude that multi-ingredient supplementation of specific antioxidants, vitamins, and plant extracts may be efficacious for improving fertility in overweight and obese couples and should be considered as a viable adjunctive treatment in future clinical trials.

## Figures and Tables

**Table 1:** Effects of the ‘Fertility Enhancer’ supplement on food consumption, bodyweights, body composition index, tissue weights, and blood glucose in Western diet-fed C57BL/6J mice.

	MALE			FEMALE		
	LF	HF	FE	LF	HF	FE
**Food Intake**						
g/day	3.4 ± 0.07	3.1 ± 0.05 *	**2.5 ± 0.17 §**	3.0 ± 0.02	2.6 ± 0.04 *	**2.2 ± 0.03 §**
kcal/day	12.3 ± 0.24	14.1 ± 0.22 *	**11.2 ± 0.74 §**	10.6 ± 0.08	12.0 ± 0.20 *	**9.8 ± 0.15 §**
**Body Weight**						
BW (g)	38.0 ± 0.9	45.8 ± 0.9 *	**39.7 ± 1.5 §**	27.1 ± 0.8	31.8 ± 1.0 *	**24.1 ± 0.4 §**
**Body Composition (NMR)**						
Fat mass (g)	11.8 ± 1.0	23.0 ± 0.7 *	**17.0 ± 1.7 §**	5.0 ± 1.3	13.2 ± 1.7 *	**4.1 ± 1.1 §**
Fat mass (%)	34.3 ± 2.3	52.2 ± 1.2 *	**46.5 ± 3.1 §**	19.7 ± 3.7	42.4 ± 3.7 *	**16.4 ± 3.6 §**
Body Composition Index	1.93 ± 0.15	1.10 ± 0.04 *	**1.31 ± 0.12 §**	4.05 ± 0.61	1.50 ± 0.19 *	**5.47 ± 1.2 §**
**Tissue Weights**						
Perirenal WAT (mg)	438.1 ± 86.4	882.6 ± 34.8 *	**602.5 ± 137.0 §**	229.4 ± 102.5	465.1 ± 105.8 *	**115.1 ± 65.2 §**
Mesenteric WAT (mg)	403.8 ± 52.8	963.25 ± 99.4 *	**606.8 ± 129.9 §**	194.8 ± 57.1	315.0 ± 47.3 *	**92.6 ± 31.8 §**
Perigonadal WAT (mg)	1538.6 ± 130.7	2670.3 ± 48.3 *	2532.7 ± 233.9	475.0 ± 117.8	1290.2 ± 191.2 *	**301.7 ± 81.4 §**
Inguinal WAT (mg)	313.4 ± 31.2	462.8 ± 55.0 *	**331.8 ± 48.8 §**	168.9 ± 42.0	478.7 ± 54.9 *	**99.8 ± 17.9 §**
Total WAT (mg)	3367 ± 376	6223 ± 297 *	**5092 ± 560 §**	1335 ± 399	3186 ± 499 *	**762 ± 245 §**
Liver (mg)	1328.9 ± 80.4	3176.4 ± 153.5 *	**1354.6 ± 83.2 §**	951.6 ± 89.0	1553.1 ± 124.2 *	**1014.4 ± 17.8 §**
**Blood Glucose**						
Fasting (mmol/L)	9.9 ± 1.2	12.5 ± 0.6 *	11.0 ± 0.8	9.20 ± 0.6	10.6 ± 0.3	**7.1 ± 0.5 §**
AUC	1819.9 ± 128.3	2084.3 ± 168.8	2144.5 ± 128.5	1683.9 ± 157.3	2028.4 ± 123.5	1674.4 ± 83.1

LF = Low Fat Diet, HF = Western diet (WD), and FE = ‘Fertility Enhancer’. Total WAT is the sum of mesenteric, inguinal, peri-renal, and gonadal depots. The body composition index is total lean mass divided by total fat mass assessed by NMR. * Significantly different from LF (*p* ≤ 0.05). § Significantly different from HF (*p* ≤ 0.05).

**Table 2:** Effect of multi-nutrient supplementation on mating behavior and reproductive outcomes in Western diet-fed C57BL/6J mice.

Mating and Reproductive Outcomes	LF	HF	FE
Copulatory plugs (5-day total)	10	1 *	6
Copulatory plugs (% total females)	50%	6%	30%
Number of litters (total)	7	0 *	4
Copulatory success (%; Litters/Plugs)	70%	0%	67%

Harem-breeding of mice occurred in a 1:2 male-to-female ratio during a five-day period (LF: *n* = 20 females, HF: *n* = 18 females, and FE: *n* = 20 females). Vaginal plugs were counted at six a.m. each morning and females were singly housed following mating. LF = Low Fat Diet, HF = Western Diet (WD), and FE = ‘Fertility Enhancer’ multi-nutrient supplement with WD. * Significantly different from LF (*p* ≤ 0.05).

**Table 3:** Effect of multi-nutrient supplementation on gonadal morphology and pathology in a subset of Western diet-fed C57BL/6J mice.

	MALE			FEMALE		
	LF (*n* = 5)	HF (*n* = 5)	FE (*n* = 5)	LF (*n* = 3)	HF *(n* = 3)	FE (*n* = 3)
**Gonadal pathology**						
**Testicular pathology**						
Mass (mg)	189.5 ± 3.1	242.0 ± 9.5 *	**199.1 ± 8.4 §**	NA	NA	NA
Seminiferous tubule diameter (AU; pixels)	571.1 ± 10.0	604.7 ± 12.6	568.5 ± 24.0	NA	NA	NA
Seminiferous epithelia (pathology score)	0.9 ± 0.5	0.7 ± 0.2	0.5 ± 0.2	NA	NA	NA
Seminiferous tubules with apoptosis (%)	29.5 ± 1.7	38.1 ± 17.8	28.6 ± 5.4	NA	NA	NA
**Ovarian pathology**						
Mass (mg)	NA	NA	NA	16.0 ± 1.0	19.4 ± 0.9	17.4 ± 1.0
Volume (mm^3^)	NA	NA	NA	0.046 ± 0.013	0.054 ± 0.010	0.040 ± 0.007
Viable follicles (#)	NA	NA	NA	61.7 ± 10.3	55.7 ± 19.9	55.7 ± 17.6
Viable follicles (#/mm^3^)	NA	NA	NA	1574.4 ± 465.6	986.1 ± 296.5	1344.5 ± 301.7
Growing follicles (#)	NA	NA	NA	29.0 ± 2.9	23.0 ± 7.9	29.0 ± 8.7
Growing follicles (#/mm^3^)	NA	NA	NA	754.5 ± 209.2	402.9 ± 73.9	691.6 ± 134.6
Atretic follicles (#)	NA	NA	NA	18.0 ± 1.5	26.0 ± 1.0 *	**12.0 ± 1.0 §**
Atretic follicles (#/mm^3^)	NA	NA	NA	461.6 ± 128.5	512.8 ± 85.0	317.6 ± 52.1
Apoptotic granulosa cells (#/ovary)	NA	NA	NA	424.0 ± 109.1	606.5 ± 321.1	364.2 ± 81.6
Apoptotic granulosa cells (#/follicle)	NA	NA	NA	15.1 ± 5.7	15.6 ± 7.5	14.5 ± 2.9

Seminiferous epithelia pathology and ovarian follicular stages assessed in hematoxylin and eosin-stained paraffin cross-sections (200× total magnification; Image J Software). Apoptotic cells were counted in seminiferous tubules and ovarian follicles following TUNEL-staining (200×). Seminiferous epithelia were scored as 0 (healthy), 1 (mild), 2 (moderate), or 3 (severe) pathology. Viable follicles = all follicles counted excluding atretic and corpora lutea. Growing follicles = all follicles counted excluding primordial, atretic, and corpora lutea. Follicles containing dark, pyknotic nuclei within the granulosa cells were considered atretic. LF = Low Fat Diet, HF = Western diet (WD), and FE = ‘Fertility Enhancer’ multi-nutrient supplement with WD. * Significantly different from LF (*p* ≤ 0.05). § Significantly different from HF (*p* ≤ 0.05).

## Data Availability

All data and statistical analyses are available upon reasonable request to the first or senior author of this manuscript.

## Supplementary Materials

The following supporting information can be downloaded at: https://www.mdpi.com/article/10.3390/nu15030611/s1, Figure S1: Pair-feeding experiment comparing lipolytic effects of the weight loss-driving ingredients in the Fertility Enhancer diet (T7) vs. calorie-matched, pair-fed controls (PT7) in high fat-fed C57 mice. Table S1: Ovarian and testicular messenger RNA levels.
