# Supplementary material for: A Multi-Ingredient Supplement Protects against Obesity and Infertility in Western Diet-Fed Mice
Source: Nutrients. 2023 Jan 25;15(3):611. doi: 10.3390/nu15030611 (PMC9921271; doi:10.3390/nu15030611)
Supplement: Supplementary file 1 [file nutrients-15-00611-s001.zip › nutrients-2090193-supplementary.pdf]

Supporting information:

Figure S1. Pair-feeding experiment comparing lipolytic effects of the weight loss-driving ingredients in the Fertility Enhancer diet (T7) vs. calorie-matched, pair-fed controls (PT7) in high fat-fed C57 mice.

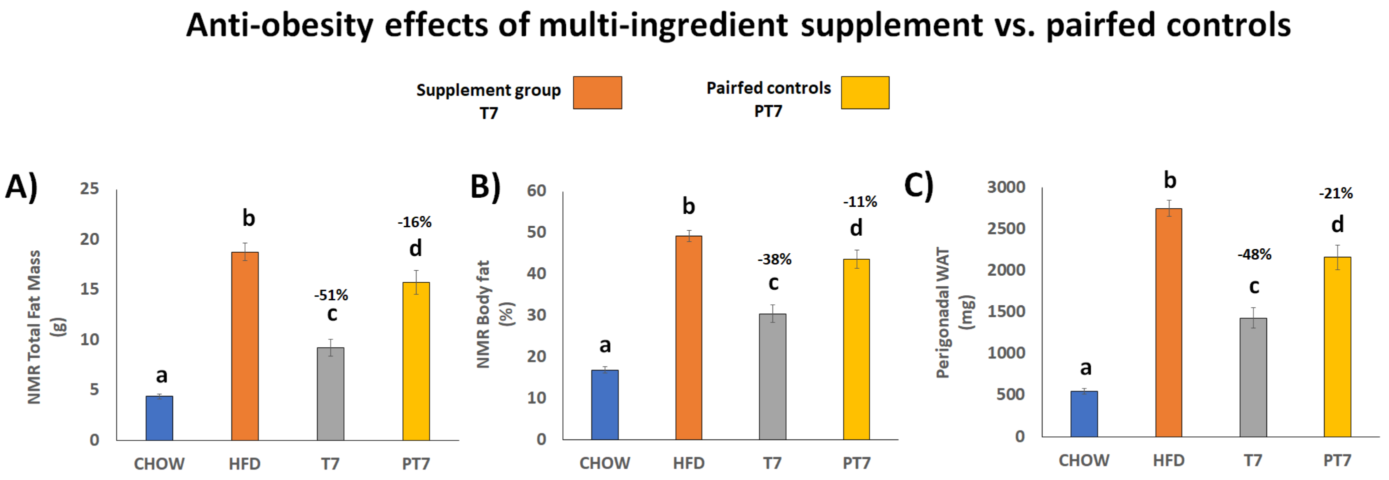

Figure S1. A). Total fat mass from NMR scan. B). Percent body fat from NMR scan. C). Perigonadal white adipose tissue mass from tissue harvest. CHOW = low fat-fed, HFD = high fat-fed, T7 = HFD + weight loss-driving ingredients from the Fertility Enhancer, and PT7 = Calorie-matched, pair-fed control group to T7. The caloric intakes of the groups were 12.42 kcals/day for CHOW, 15.21 kcals/day for HFD, and 11.63 kcals/day for T7, with the pair-fed PT7 group receiving the same daily calories as T7. As demonstrated, appetite suppression accounted for 30-40% of the observed weight loss in animals receiving the T7 multi-ingredient supplement (e.g., alpha lipoic acid, coQ10, vitamin E, green tea extract, green coffee bean extract, forskolin extract, and beetroot extract). Groups with different letters are significantly different ($p < 0.05$)

Table S1. Ovarian and testicular messenger RNA levels.

	LF	HF	FE	One-way ANOVA
**Ovarian mRNA**				
**Fold change ($2^{-\Delta\Delta CT}$)**				
TNF-a	1.1 ± 0.2	1.3 ± 0.4	0.7 ± 0.2	NS
IL-1b	1.1 ± 0.2	**3.3 ± 1.1***	**0.7 ± 0.2§**	**p = 0.023**
Casp 1	0.8 ± 0.1	**2.0 ± 0.4***	1.1 ± 0.3	**p = 0.033**
p21	1.1 ± 0.2	1.6 ± 0.4	1.6 ± 0.3	NS
Casp 3	1.0 ± 0.1	1.1 ± 0.2	1.1 ± 0.1	NS
Casp 9	1.0 ± 0.1	1.2 ± 0.4	1.1 ± 0.3	NS
ND1	1.1 ± 0.2	0.8 ± 0.1	0.7 ± 0.1	NS
SDHA	1.1 ± 0.1	0.8 ± 0.1	1.0 ± 0.1	NS
**Testicular mRNA**				
**Fold change ($2^{-\Delta\Delta CT}$)**				
TNF-a	1.1 ± 0.4	2.1 ± 0.3	2.9 ± 1.0	NS
IL-1b	1.0 ± 0.1	1.6 ± 0.2	1.7 ± 0.3	NS
CD86	1.1 ± 0.2	1.7 ± 0.2	1.7 ± 0.3	NS
IL6	1.2 ± 0.4	1.7 ± 0.3	2.1 ± 0.8	NS
NLRP3	1.4 ± 0.6	2.5 ± 1.0	2.3 ± 0.7	NS
Casp 1	1.0 ± 0.1	1.6 ± 0.2	1.7 ± 0.5	NS
Casp 3	1.0 ± 0.1	1.3 ± 0.01	1.4 ± 0.3	p = 0.06
Casp 9	1.0 ± 0.1	1.4 ± 0.1	1.8 ± 0.3	p = 0.07
PARP1	1.0 ± 0.1	1.4 ± 0.3	1.6 ± 0.2	NS
Srebp2	1.0 ± 0.2	1.3 ± 0.1	1.3 ± 0.2	NS
AR	1.0 ± 0.1	**1.5 ± 0.2***	1.4 ± 0.1	**p < 0.01**
ApoA1	1.1 ± 0.3	1.7 ± 0.8	0.8 ± 0.2	NS
Lepr	1.1 ± 0.2	2.0 ± 0.2	1.9 ± 0.6	NS

*Significantly different from LF ($p \leq 0.05$). § Significantly different from HF ($p \leq 0.05$).
